# Development of novel echogenic-imageable thermosensitive liposome for optimizing tumor drug distribution using ultrasound guided HIFU

**DOI:** 10.1186/2050-5736-3-S1-P73

**Published:** 2015-06-30

**Authors:** Ashish Ranjan, Danny Maples, Ryan Newhardt, Venkatesan Perumal

**Affiliations:** 1 Oklahoma State University, Stillwater, Oklahoma, United States

## Background/introduction

A major challenge in HIFU mediated Image Guided Drug Delivery (IGDD) is developing accurate means to implement real-time drug delivery control, and to optimize intratumoral drug distribution. To facilitate clinical translation, objectives of this study were to: 1) develop echogenic E-LTSL, a low temperature sensitive liposome co-loaded with an US contrast agent (Perfluoropentane, PFP) and doxorubicin, 2) determine stability of contrast agent encapsulation and characterize doxorubicin release from E-LTSL and 3) investigate the ability of E-LTSL to report on real-time doxorubicin distribution with Ultrasound (US)-guided hyperthermia.

## Methods

E-LTSL was loaded passively with PFP using an innovative 1-step sonoporation method and actively loaded with doxorubicin. Doxorubicin release and PFP imageability from E-LTSL in phantoms was quantified by fluorescence spectroscopy, ultrasound imaging and transmission electron microscopy (TEM) in combination with mild hyperthermia (40-42°C). Ultrasound imageability of E-LTSL was determined *in vivo* in a mouse xenograft model of human prostate cancer.

## Results and conclusions

TEM images confirmed that the PFP emulsion formation is contained within LTSL. Phantom study clearly showed that only E-LTSLs are echogenic. Temperature *vs*. size increase and drug release kinetics of E-LTSL demonstrated no difference with control. Doxorubicin release in physiological buffer was <5% in 1 hr at baseline (25°C) and body temperatures (37°C), *vs*. >99% release with hyperthermia (~41°C). Intensity of observed ultrasound image with respect to temperature in the range of 31-40°C correlated strongly to the formation of gas bubbles in E-LTSL, and stabilized to a fixed intensity at the transition temperature. After the transition temperature of E-LTSL reached, the US intensity increased again similar to Dox release. Synthesized E-LTSLs were imageable *in vivo* and were stable in aqueous environment, with no visual evidence of particle aggregation after 48 hr storage at 4 °C. In conclusion, an US imageable heat sensitive liposome formulation co-loaded with doxorubicin and an US contrast agent was developed. Stability, imageability, and US monitoring of contrast agent and Dox release suggest that US-guided drug delivery from E-LTSL may assist physicians in real-time tumor drug delivery mapping. *In vivo* US-guided HIFU in combination with E-LTSL to demonstrate enhanced tumor drug distribution in C26 colon cancer model is currently in progress. This technology has potential for clinical translation.

